# Does overexpression of HER-2 correlate with clinicopathological characteristics and prognosis in colorectal cancer? Evidence from a meta-analysis

**DOI:** 10.1186/s13000-015-0380-3

**Published:** 2015-08-16

**Authors:** Sheng-wen Wu, Cong-chao Ma, Wen-hui Li

**Affiliations:** Department of General Surgery, The Affiliated Jianhu Hospital of Nantong University, Jianhu People’s Hospital, Jianhu, 224700 Jiangsu Province China; Department of Interventional Radiology, The Affiliated Yancheng Hospital of Southeast University Medical College, Yancheng Third People’s Hospital, Yancheng, 224001 Jiangsu Province China

## Abstract

**Background:**

Previous studies have been inconsistent with respect to the reported associations between human epidermal growth factor receptor (HER-2/neu) overexpression in colorectal cancer. The aims of this meta-analysis are to assess its correlation with clinicopathological characteristics and prognostic significance in colorectal cancer.

**Methods:**

Eligible studies were searched in Pubmed, Embase and Web of Science databases. The inclusion criteria were studies that assessed the relationship between HER-2 expression detected by immunohistochemistry (IHC) and the prognosis or clinicopathological features in patients with colorectal cancer (CRC). Subgroup analysis according to sex, tumor location, TNM stage, grade of differentiation and lymph node metastasis were produced. Odds ratio (OR) or hazard ratio (HR) with 95 % confidence interval (CI) were calculated to examine the risk or hazard association, and heterogeneity and publication bias analyses were also performed.

**Results:**

A total of 18 studies comprising 2867 colorectal cancer patients were included to assess the association between HER-2 immunohistochemical expression and clinicopathological characteristics and survival. The overall analysis showed that there was no detectable relation between HER-2 expression and prognosis in colorectal cancer patients with the pooled HR of 1.08 (95 % CI: 0.96–1.21, *P* = 0.21). With respect to clinicopathological features, there was also no detectable relation between HER-2 expression and sex (OR = 0.91, 95 % CI: 0.72–1.15, *P* = 0.42), tumor location (OR = 1.21, 95 % CI = 0.88–1.65, *P* = 0.24), grade of differentiation (OR = 1.03, 95 % CI = 0.72–1.47, *P* = 0.86), TNM stages (OR = 0.72, 95 % CI = 0.31–1.66, *P* = 0.44), or lymph node metastasis (OR = 1.90, 95 % CI = 0.90–4.02, *P* = 0.09) in CRC.

**Conclusions:**

The finding from this present meta-analysis suggested that HER-2 overexpression was not related to clinicopathological characteristics and poor prognostic of colorectal cancer patients.

## Background

Despite the clinical prognosis for colorectal cancer has been improved by the development of surgery and adjuvant chemoradiotherapy, the long-term survival of colorectal cancer patients is highly unsatisfactory and hindered by recurrence and distant metastasis [1]. The prognostic factors that have been implicated include demographics, tumor size, tumor site, stage, and response to chemotherapy. However, the mechanism of prognosis in colorectal cancer patients is still not fully understood. Therefore, a better understanding into its basic biology is urgently needed to identify its prognostic markers and therapeutic targets. Much effort has been made for a long time to identify an established marker possessing the predicative value for survival of colorectal cancer patients remains a topic that needs to be explored.

Human epidermal growth factor (HER-2/neu), which encodes a 185-kDa transmembrane tyrosine kinase [2] and overexpression of HER-2 gene has been found to correlate with poor prognosis in a variety of human cancers, such as breast, ovarian, and lung cancers [3]. For instance, overexpression of the HER-2/neu is detectable in 25–35 % of breast cancers [4]. Treatment of these patients with trastuzumab (Herceptin), an anti-HER-2/neu monoclonal antibody, has been shown to reduce tumor volume, magnify the effects of chemotherapy, and increase survival rate in primary and metastatic breast cancer [5]. However, there is still a lack of general consensus regarding the possible prognostic impact of HER-2 in colorectal cancer.

Although a large number of studies were performed on patients with colorectal cancer, the prognostic value of HER-2 expression for colorectal cancer patients remains controversial. We thus conducted this meta-analysis to indicate the correlation between HER-2 expression and the clinicopathological characteristics of colorectal cancer and estimate whether HER-2 can act as a prognostic marker for patients with colorectal cancer.

## Methods

### Literature search

Pubmed, Embase and Web of Science were systematically searched. The search ended in September 1st, 2014, and no lower date limit was used. Bibliographies cited in an identified article were also searched manually to triage other suitable studies. No language restriction was applied, and we also screened the references of the relevant studies to check for potentially relevant articles. The search strategy included the following keywords variably combined by “human epidermal growth factor receptor 2”, “HER-2/neu”,“c-erbB2”, “colorectal cancer”, “survival” and “prognosis”. Internet search engines were also used to perform a manual search for abstracts from international meetings, which were then downloaded and studied.

### Inclusion and exclusion criteria

Criteria for eligibility of a study included in this meta-analysis were: (1) HER-2 expression was measured by immunohistochemistry, Western blot or fluorescence in situ hybridization; (2) overall survival rates between different expressions of HER-2 in colorectal cancer were compared and (3) hazard ratios (HR) for overall survival rates according to HER-2 expression were reported or could be calculated from the data presented. When several studies were reported from the same authors or organizations, the meta-analysis enrolled the most recent or highest quality study only if the most recent one did not fit the inclusion criteria. Studies were excluded if (1) Studies were case reports, letters, and reviews without original data; animal or laboratory studies; (2) the samples came from lymph nodes or the peritoneal cavity; (3) the outcomes of interest were not reported and it was impossible to calculate outcomes from the originally published data or (4) repeated studies were based on the same database or patients. To avoid the influence of redundant studies, we checked all of the authors and organizations, and evaluated the accrual period and community of patients enrolled for each study.

### Data extraction

Extracted data were crosschecked between the two authors (SWW and CCM) to rule out any discrepancy. Data regarding the following for each included studies were extracted independently: first authors’ surname, publication year, sample size, HER-2 assessment methods, the cut-off definition, clinicopathological characteristics, and data of HER-2 overexpression for the disease-specific or overall survival. For the survival analysis, the HR, which takes into account the number and timing of events, is the most appropriate statistic to analyze time-to-event outcomes [6]. For each study the HR and its 95 % confidence intervals (CIs) were estimated depending upon the data provided in the literature. In some studies, the HR could be obtained directly from publications, or could be calculated by using the O-E statistic and variance in the manuscript, and unadjusted HRs were considered preferable to multivariable adjusted HRs in these cases. If a directly reported HR was not available, HR estimates were calculated from the data including the number of patients at risk in each group, the total number of events and the log-rank statistic or its p value. Otherwise, we extracted survival rates at specified times from Kaplan-Meier survival curves to reconstruct the HR estimate according to previously described methods [7], with the assumption that patients were censored at an unchanged rate during the follow-up. Disagreements were discussed by the authors and resolved by consensus.

### Statistical analysis

The statistical analysis was carried out using the Review Manager 5.1. HR was used in this time-to-event analysis, which takes into consideration the number and timing of events. The disease-specific or overall survival value of each study was determined by the combination of HR and its corresponding 95 % CI. All data were directly obtained or indirectly computed using the formula recommended by Tierney [7]. The significance of the pooled HR was determined by Z test, and P ≤ 0.05 was considered statistically significant. Pooled HR was calculated using a fixed-effects model or random-effects model to evaluate the relationship between HER-2 overexpression and the disease-specific or overall survival. I^2^ statistics was used to evaluate the between-study heterogeneity analysis in this meta-analysis [8]. The random-effects model was used when an obvious heterogeneity was observed among the included studies (*I*^*2*^ > 50 %). The fixed effects model was used when there was no significant heterogeneity between the included studies (*I*^*2*^ ≤ 50 %). Meta-regression analysis by stratifying on study location, publication year, number of patients, quality score and cut-off value were conducted. For the pooled analysis of the correlation between HER-2 overexpression and clinicopathological features (sex, tumor location, TNM stage, grade of differentiation, lymph node metastasis), odds ratios (ORs) and 95 % CIs were combined to estimate the effect. An observed HR or OR > 1 implied a worse prognosis for the group with HER-2 overexpression and would be considered to be statistically significant if the 95 % CI did not overlap. Publication bias was estimated using a funnel plot with a Begg’s test; funnel plot asymmetry on the natural logarithm scale of the HR was measured by a line arregression approach.

## Results

### Study selection and characteristics

**Fig. 1:**
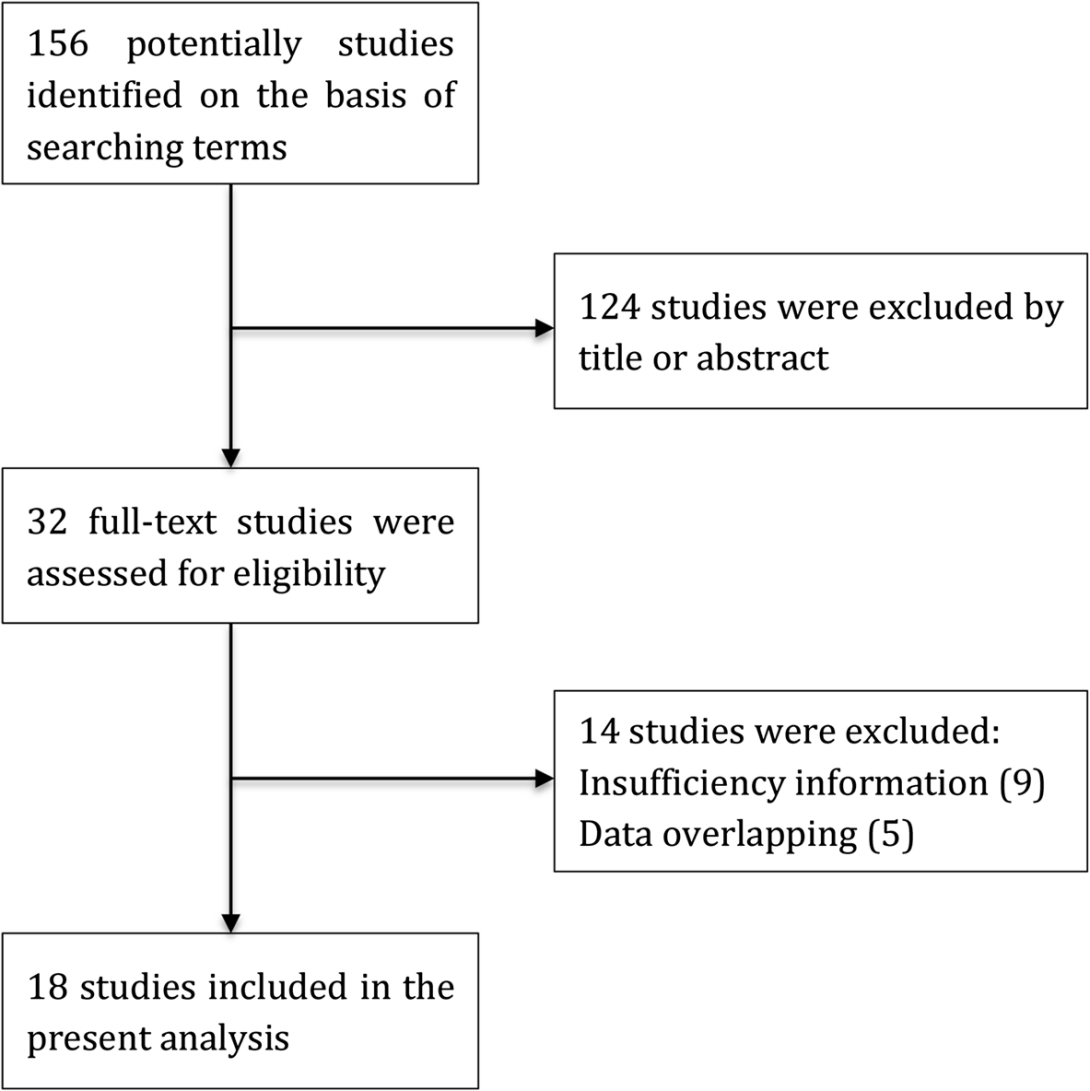
Flow diagram of studies selection procedure

A total of 156 articles were identified from a search of the above databases using the search strategy as described above. After exclusion of the trials that were out of the scope of our meta-analysis, 32 studies assessing prognostic value for survival of HER-2 status in patients with colorectal cancer were considered eligible for inclusion in the evaluation. Upon further review, 9 were excluded because it was not possible to allow for the calculation of HR estimate because of insufficient reported data, 5 were excluded because they have overlapped data with other studies. Finally, a total of 18 publications [9–26] were enrolled for the present meta-analysis (Fig. 1).

The clinical characteristics of these 18 included studies eligible for the meta-analysis are summarized in Table 1. Three study assessed the patients from China, three from Korea, two from Japan, one from Germany, one from Egypt, one from Switzerland, two from Brazil, one from Ireland, two from Greece, one from USA and one from UK. The total number of patients included was 2867, ranging from 44 to 355 patients per study. All of the included studies used IHC to determine HER-2 status. In each study, the IHC cut-off values of HER-2 appeared to be different. These eligible studies were published from 1995 to 2014.

**Table 1:** Characteristics of studies included in the meta-analysis

Study	Year	Country	No. of patients	Age (year)	Technique	Cut-off value	No. of HER-2 Positive (%)	Follow-up period (month)	Outcome
Baiocchi	2009	Brazil	109	NA	IHC	>10 %	Positive (%)	57.4 (median)	OS
Chen	2010	China	44	55 (median)	IHC	1+	14 (31.8)	35 (median)	OS, CP
Conradi	2013	Germany	264	63 (mean)	IHC, S-ISH	2+	70 (26.5)	48.5 (median)	OS
Drecoll	2014	Switzerland	355	66 (median)	IHC, FISH	2+	51 (14.4)	87 (mean)	OS
Ishida	2000	Japan	149	61.6 (mean)	IHC	NA	44 (29.5)	NA	OS, CP
Ismail	2007	Egypt	104	47.4 (mean)	IHC	1+	10 (9.6)	18 (median)	OS, CP
Jesus	2005	Brazil	108	63.1 (mean)	IHC	>10 %	52 (48.1)	28.1 (mean)	OS
Kavanagh	2009	Ireland	132	67.0 (mean)	IHC, FISH	2+	18 (13.6)	NA	OS
Lazaris	1995	Greece	60	68.75 (mean)	IHC	>10 %	21 (35)	60 (median)	OS, CP
Li	2011	China	317	57.8 (mean)	IHC	2+	49 (15.5)	68.8 (median)	OS, CP
Lim	2013	South Korea	95	69 (median)	IHC, FISH	2+	23 (24.2)	22.7 (median)	OS, CP
Lu	2012	China	126	NA	IHC	>25 %	59 (46.8)	NA	OS, CP
Mckay	2002	UK	248	NA	IHC	2+	203 (81.9)	43 (median)	OS, CP
Osako	1998	Japan	146	65.0 (mean)	IHC	NA	100 (68.5)	55.2 (median)	OS, CP
Pappas	2013	Greece	51	70.9 (mean)	IHC	2+	2 (3.9)	18 (median)	OS, CP
Park	2007	South Korea	137	62.4 (mean)	IHC, FISH	2+	65 (47.4)	48.5 (median)	OS, CP
Rossi	2002	USA	156	75 (median)	IHC	1+	24 (15.4)	46.8 (median)	OS
Shin	2014	Korea	266	63 (mean)	IHC	2+	80 (30.1)	NA	OS, CP

*IHC* immunohistochemistry, *S-ISH* silver in situ hybridization, *FISH* fluorescence in situ hybridization, *NA* not applicable, *OS* overall survival, *CP* Clinicopathological parameters

### HER-2 overexpression and OS in patients with CRC

**Fig. 2:**
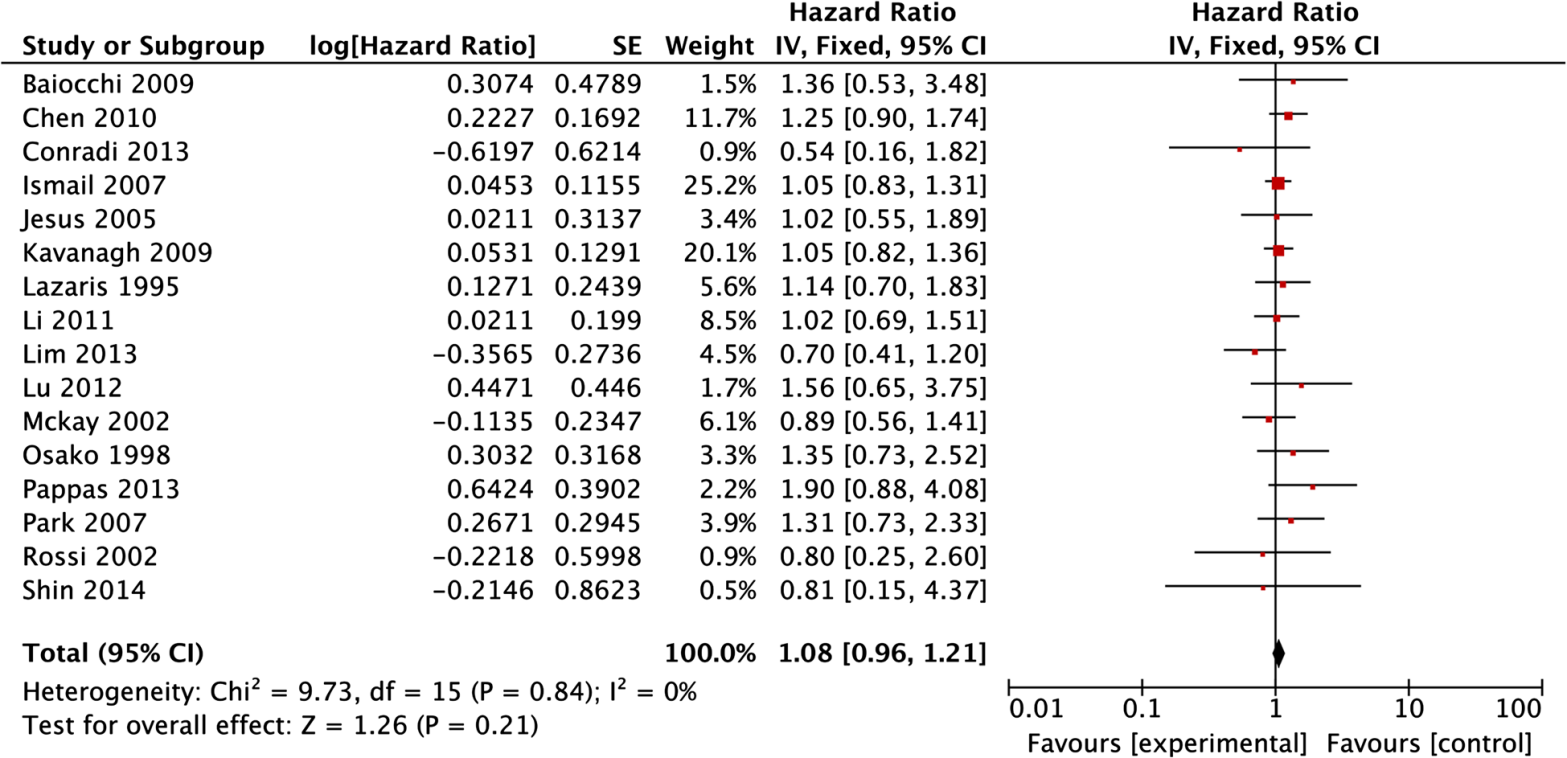
Meta-analysis of the association between HER-2 overexpression and the overall survival rate for colorectal cancer patients

Data of overall survival extracted from 16 eligible studies were included in the meta-analysis. Since the heterogeneity among those studies for HER-2 expression was insignificant (*I*^2^ = 0 %), a fixed-effect model was used to calculate the pooled HR with corresponding 95 % CI of OS in CRC patients. Meta-analysis found that there was no detectable relation between HER-2 expression and prognosis in CRC patients with the pooled HR of 1.08 (95 % CI: 0.96–1.21, *P* = 0.21) (Fig. 2).

### HER-2 overexpression and clinicopathological parameters in patients with CRC

**Fig. 3:**
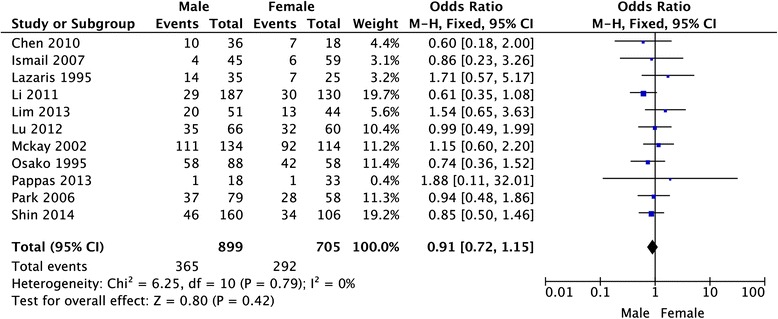
Meta-analysis of the association between HER-2 overexpression and sex for colorectal cancer patients

**Fig. 4:**
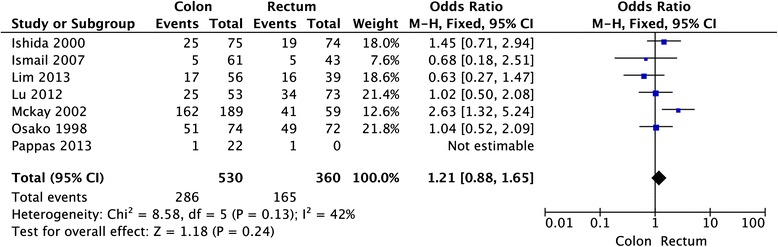
Meta-analysis of the association between HER-2 overexpression and tumor location for colorectal cancer patients

**Fig. 5:**
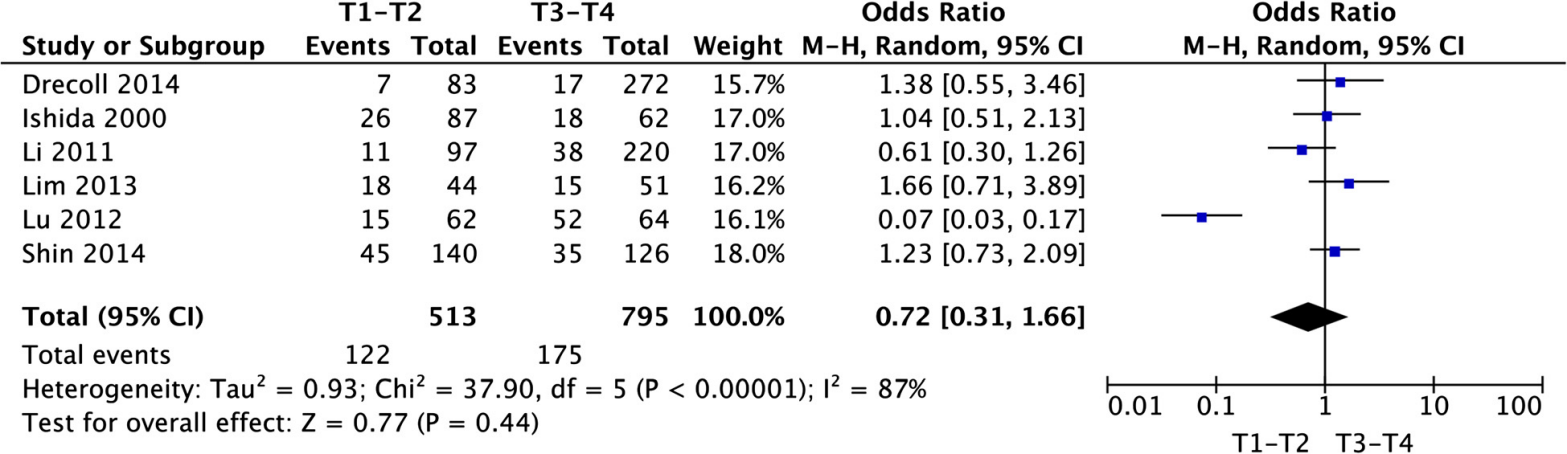
Meta-analysis of the association between HER-2 overexpression and TNM stage for colorectal cancer patients

**Fig. 6:**
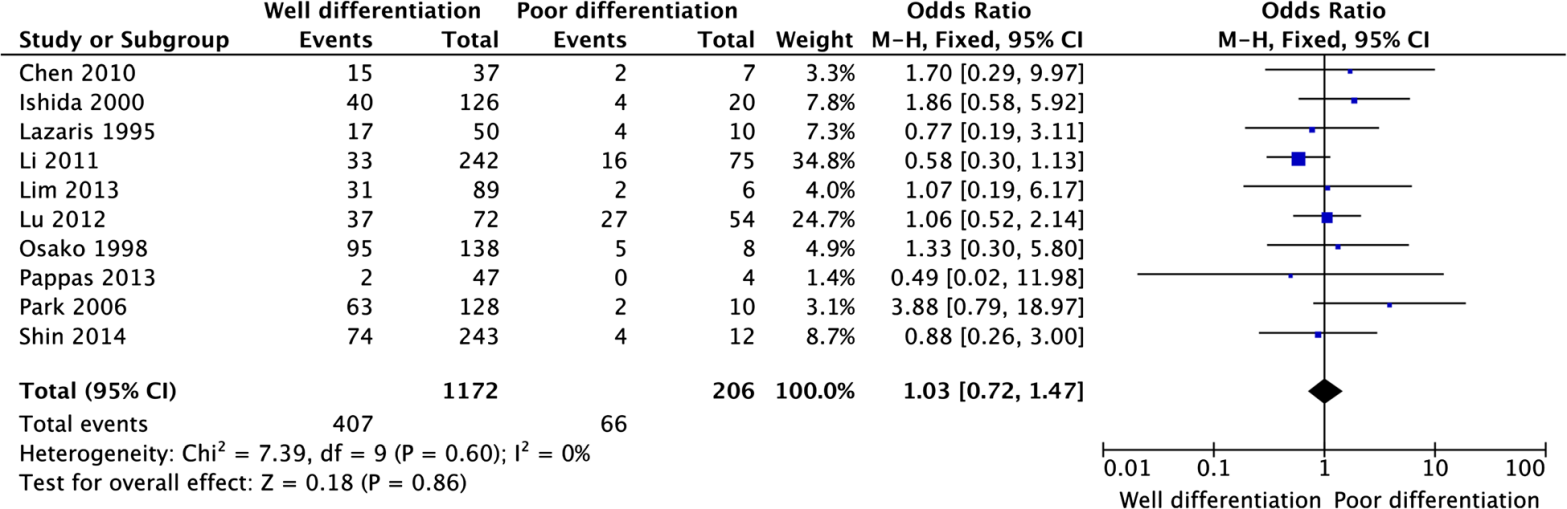
Meta-analysis of the association between HER-2 overexpression and grade of differentiation for colorectal cancer patients

**Fig. 7:**
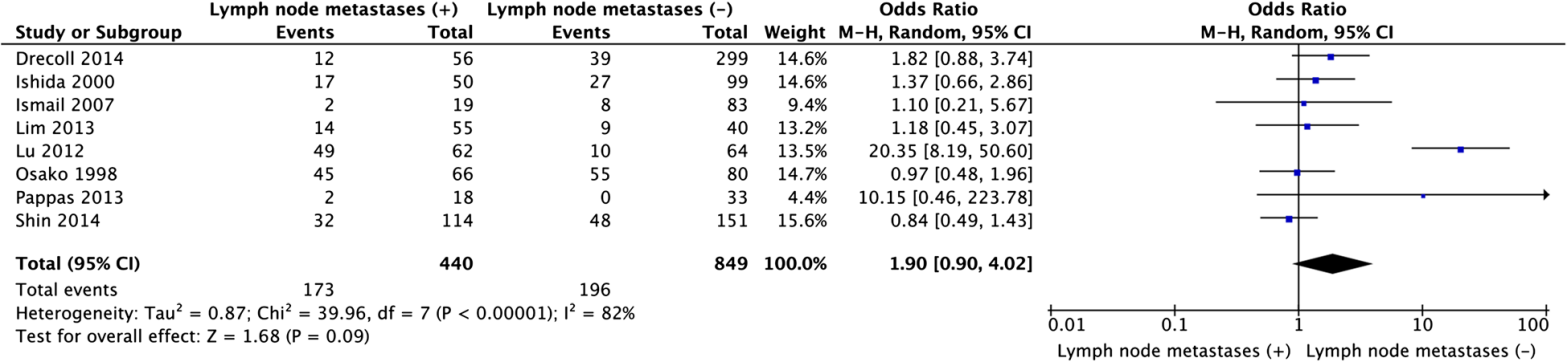
Meta-analysis of the association between HER-2 overexpression and lymph node metastasis for colorectal cancer patients

Twelve studies provided the information of clinicopathological parameters and their correlation with HER-2 positive expression was summarized in Table 1. Eleven studies demonstrated that there was no correlation between HER-2 expression and gender (OR = 0.91, 95 % CI: 0.72–1.15, *P* = 0.42, Fig. 3). Except this above-mentioned parameter, controversies also existed on the correlation among tumor location, differentiation, lymph node metastasis and HER-2 overexpression in these included studies. Seven studies evaluated the relationship between HER-2 expression and tumor location in CRC patients. The pooled OR was (OR = 1.21, 95 % CI = 0.88–1.65, *P* = 0.24, Fig. 4), suggesting that HER-2 overexpression was not associated with tumor location. In addition, no correlation was found between the overexpression of HER-2 and TNM stage (OR = 0.72, 95 % CI = 0.31–1.66, *P* = 0.44, Fig. 5), grade of differentiation (OR = 1.03, 95 % CI = 0.72–1.47, *P* = 0.86, Fig. 6). Moreover, the meta-analysis suggested that HER-2 overexpression was also not correlated with lymph node metastasis (OR = 1.90, 95 % CI = 0.90–4.02, *P* = 0.09) (Fig. 7).

### Publication bias

Begg’s funnel plot and Egger’s test were performed to assess the publication bias of studies. The results showed that no publication bias was detected in all comparisons. The shape of the funnel plot was symmetrical for the all comparisons in patients with CRC (Figures not shown).

## Discussion

Up to date, clinically approved biomarkers have been found to guide treatment and predict outcomes in several solid tumors, and several studies have demonstrated that HER-2 is a molecular prognostic biomarker of breast cancer [3, 4]. However, the clinical value of HER-2 remains controversial in several solid tumors including colorectal cancer. In this meta-analysis, we revealed that overexpression of HER-2 was negatively correlated with the poor prognosis of CRC patients. In addition, no correlation was observed between HER-2 expression and clinicopathological features including tumor location, grade of differentiation, TNM stages and lymph node metastasis.

The HER-2/neu gene is located on chromosome 17q21 and encodes a 185-kDa transmembrane protein which exhibits tyrosine kinase activity [27]. The HER-2/neu protein is extensively homologous and related to the epidermal growth factor receptor (EGFR) [28]. Similar to EGFR, the HER-2/neu protein is involved in normal cell proliferation and tissue growth. Originally, it was observed that transfection of multiple copies of the HER-2/neu gene into nonneoplastic human breast cell lines led to increased production of the HER-2/neu protein as well as malignant transformation [29]. HER-2/neu has attracted considerable attention in breast cancer, where it has been targeted successfully in the treatment of patients with advanced disease [30]. As a prognostic marker, HER-2/neu is used to predict the probable course and outcome of the disease. As a predictive marker, HER-2/neu is used to forecast the patient’s therapeutic response to adjuvant chemotherapy and endocrine therapy and to select patients for anti-HER-2/neu monoclonal antibody (Herceptin) immunotherapy. Favorable clinical results with anti-HER-2/neu antibodies in breast cancer have led to the analysis of HER-2/neu expression in other solid tumors. HER-2/neu amplification and/or overexpression has also been detected in ovarian, lung, gastric, and colon carcinomas. With regard to colorectal carcinomas, several immunohistochemical (IHC) studies have reported different frequencies of HER-2/neu overexpression, in a wide range from 0 to 30 % [31]. There are, however, conflicting results in studies of HER-2/neu with regard to its relationship to prognosis in colorectal cancer patients. Some studies have reported an association between HER-2/neu overexpression and advanced stage, decreased survival, or both. Other studies have failed to find any association with prognosis whatsoever.

A previously meta-analysis had been performed to examine the prognostic role in patients with CRC, and the result was consisted with us [32]. However, our study showed the following advancements when compared with previous work. Firstly, our study included larger sample size than previous one. After their work published, additional seven studies including 1080 patients were published [12–14, 17, 19, 23, 26]. In some degree, our results were more robust and reliable than previous work. Secondly, various subgroup analyses were done in our analysis, while in previous studies, they only conducted subgroup analysis by race. Thirdly, our study provided more information and gave a comprehensive insight on the role of HER-2 in the progression of CRC. In Li’s study, they only investigated the relationship between HER-2 overexpression and OS. However, in our study, we provided the information not only OS, but also clinicopathological features. The present study indicated that there was no detectable relation between HER-2 expression and sex, tumor location, grade of differentiation, TNM stages, or lymph node metastasis in CRC. Based on the above points, we thought our up-to date meta-analysis was worthwhile and comprehensive.

However, it should be circumspect to make a verdict of the association with HER-2 overexpression and CRC, because there are still several issues should be considered. First, the methods used for the evaluation of the levels of markers in CRC patients and the use of standard threshold, are both likely to impact on our results. In the studies we included, IHC techniques used to detect protein expression were not the same, including antibody type and concentration, the cut-off value definition and none of the included studies mention the information of interobserver variation. But it is very important for HER-2 assessing. Moreover, lack of specific criteria for assessing HER-2 positivity according to different types of cancer is a situation, which should and would be improved in the future. These differences could contribute to the heterogeneity. Second, the small size of the patient cohorts in included studies led to a lack of power in identifying the relation between the proposed prognostic factor and outcome. The studied populations may not have accurately represented the lack of abundant HER-2 expression data in global population makes it difficult to set a standard value for the measurement of HER-2 status. Third, in some studies, the HRs and their corresponding 95 % CIs were indirectly calculated from other survival data or extracted from the survival curves, which may bias our results. All of these factors might partly influence the significance of HER-2 expression in the survival and the clinicopathological analysis.

## Conclusion

This meta-analysis indicated that overexpression of HER-2 was not related to the poor survival in CRC patients and no correlation exists between HER-2 overexpression and other common clinicopathological parameters such as tumor location, TNM stage, tumor differentiation and lymph node metastasis. Therefore, HER-2/neu overexpression might not be a significantly progress and prognostic indicator for patients. Well-designed clinical studies with large cases of CRC should be performed in the future to validate the relationship between HER-2 overexpression and prognosis of CRC patients.

## References

[1] Sung JJ, Lau JY, Goh KL, Leung WK, Asia Pacific Working Group on Colorectal C (2005). Increasing incidence of colorectal cancer in Asia: implications for screening. Lancet Oncol.

[2] Hynes NE, Lane HA (2005). ERBB receptors and cancer: the complexity of targeted inhibitors. Nat Rev Cancer.

[3] Slamon DJ, Clark GM, Wong SG, Levin WJ, Ullrich A, McGuire WL (1987). Human breast cancer: correlation of relapse and survival with amplification of the HER-2/neu oncogene. Science.

[4] Slamon DJ, Godolphin W, Jones LA, Holt JA, Wong SG, Keith DE (1989). Studies of the HER-2/neu proto-oncogene in human breast and ovarian cancer. Science.

[5] Slamon D, Eiermann W, Robert N, Pienkowski T, Martin M, Press M (2011). Adjuvant trastuzumab in HER2-positive breast cancer. N Engl J Med.

[6] Parmar MK, Torri V, Stewart L (1998). Extracting summary statistics to perform meta-analyses of the published literature for survival endpoints. Stat Med.

[7] Tierney JF, Stewart LA, Ghersi D, Burdett S, Sydes MR (2007). Practical methods for incorporating summary time-to-event data into meta-analysis. Trials.

[8] Higgins JP, Thompson SG, Deeks JJ, Altman DG (2003). Measuring inconsistency in meta-analyses. BMJ.

[9] Baiocchi G, Lopes A, Coudry RA, Rossi BM, Soares FA, Aguiar S (2009). ErbB family immunohistochemical expression in colorectal cancer patients with higher risk of recurrence after radical surgery. Int J Colorectal Dis.

[10] Chen J, Li Q, Wang C, Wu J, Zhao G (2010). Prognostic significance of c-erbB-2 and vascular endothelial growth factor in colorectal liver metastases. Ann Surg Oncol.

[11] Conradi LC, Styczen H, Sprenger T, Wolff HA, Rodel C, Nietert M (2013). Frequency of HER-2 positivity in rectal cancer and prognosis. Am J Surg Pathol.

[12] Drecoll E, Nitsche U, Bauer K, Berezowska S, Slotta-Huspenina J, Rosenberg R (2014). Expression analysis of heat shock protein 90 (HSP90) and Her2 in colon carcinoma. Int J Colorectal Dis.

[13] Ishida H, Sadahiro S, Suzuki T, Ishikawa K, Tajima T, Makuuchi H (2000). c-erbB-2 protein expression and clinicopathologic features in colorectal cancer. Oncol Rep.

[14] Ismail HM, El-Baradie M, Moneer M, Khorshid O, Touny A (2007). Clinico-pathological and prognostic significance of p53, Bcl-2 and Her-2/neu protein markers in colorectal cancer using tissue microarray. J Egypt Natl Canc Inst.

[15] Jesus EC, Matos D, Artigiani R, Waitzberg AF, Goldenberg A, Saad SS (2005). Assessment of staging, prognosis and mortality of colorectal cancer by tumor markers: receptor erbB-2 and cadherins. Acta Cir Bras.

[16] Kavanagh DO, Chambers G, O’Grady L, Barry KM, Waldron RP, Bennani F (2009). Is overexpression of HER-2 a predictor of prognosis in colorectal cancer?. BMC Cancer.

[17] Lazaris AC, Theodoropoulos GE, Anastassopoulos P, Nakopoulou L, Panoussopoulos D, Papadimitriou K (1995). Prognostic significance of p53 and c-erbB-2 immunohistochemical evaluation in colorectal adenocarcinoma. Histol Histopathol.

[18] Li Q, Wang D, Li J, Chen P (2011). Clinicopathological and prognostic significance of HER-2/neu and VEGF expression in colon carcinomas. BMC Cancer.

[19] Lim SW, Kim HR, Kim HY, Huh JW, Kim YJ, Shin JH (2013). Over-expression of Her-2 in colorectal cancer tissue, but not in serum, constitutes an independent worse prognostic factor. Cell Oncol (Dordr).

[20] Lu Y, Jingyan G, Baorong S, Peng J, Xu Y, Cai S (2012). Expression of EGFR, Her2 predict lymph node metastasis (LNM)-associated metastasis in colorectal cancer. Cancer Biomark.

[21] McKay JA, Loane JF, Ross VG, Ameyaw MM, Murray GI, Cassidy J (2002). c-erbB-2 is not a major factor in the development of colorectal cancer. Br J Cancer.

[22] Osako T, Miyahara M, Uchino S, Inomata M, Kitano S, Kobayashi M (1998). Immunohistochemical study of c-erbB-2 protein in colorectal cancer and the correlation with patient survival. Oncology.

[23] Pappas A, Lagoudianakis E, Seretis C, Tsiambas E, Koronakis N, Toutouzas K (2013). Clinical role of HER-2/neu expression in colorectal cancer. J BUON.

[24] Park DI, Kang MS, Oh SJ, Kim HJ, Cho YK, Sohn CI (2007). HER-2/neu overexpression is an independent prognostic factor in colorectal cancer. Int J Colorectal Dis.

[25] Rossi HA, Liu Q, Banner B, Hsieh CC, Savas L, Savarese D (2002). The prognostic value of invariant chain (Ii) and Her-2/neu expression in curatively resected colorectal cancer. Cancer J.

[26] Shin IY, Sung NY, Lee YS, Kwon TS, Si Y, Lee YS (2014). The expression of multiple proteins as prognostic factors in colorectal cancer: cathepsin D, p53, COX-2, epidermal growth factor receptor, C-erbB-2, and Ki-67. Gut Liver.

[27] Akiyama T, Sudo C, Ogawara H, Toyoshima K, Yamamoto T (1986). The product of the human c-erbB-2 gene: a 185-kilodalton glycoprotein with tyrosine kinase activity. Science.

[28] Coussens L, Yang-Feng TL, Liao YC, Chen E, Gray A, McGrath J (1985). Tyrosine kinase receptor with extensive homology to EGF receptor shares chromosomal location with neu oncogene. Science.

[29] Di Fiore PP, Pierce JH, Kraus MH, Segatto O, King CR, Aaronson SA (1987). erbB-2 is a potent oncogene when overexpressed in NIH/3T3 cells. Science.

[30] Slamon DJ, Leyland-Jones B, Shak S, Fuchs H, Paton V, Bajamonde A (2001). Use of chemotherapy plus a monoclonal antibody against HER2 for metastatic breast cancer that overexpresses HER2. N Engl J Med.

[31] Kapitanovic S, Radosevic S, Kapitanovic M, Andelinovic S, Ferencic Z, Tavassoli M (1997). The expression of p185(HER-2/neu) correlates with the stage of disease and survival in colorectal cancer. Gastroenterology.

[32] Li C, Liu DR, Ye LY, Huang LN, Jaiswal S, Li XW (2014). HER-2 overexpression and survival in colorectal cancer: a meta-analysis. J Zhejiang Univ Sci B.

